# The Burden of Common Infectious Disease Syndromes at the Clinic and Household Level from Population-Based Surveillance in Rural and Urban Kenya

**DOI:** 10.1371/journal.pone.0016085

**Published:** 2011-01-18

**Authors:** Daniel R. Feikin, Beatrice Olack, Godfrey M. Bigogo, Allan Audi, Leonard Cosmas, Barrack Aura, Heather Burke, M. Kariuki Njenga, John Williamson, Robert F. Breiman

**Affiliations:** 1 International Emerging Infections Program-Kenya, Centers for Disease Control and Prevention-Nairobi and Kisumu, Nairobi and Kisumu, Kenya; 2 Kenya Medical Research Institute/CDC Public Health and Research Collaboration, Kisumu and Nairobi, Kenya; Walter and Eliza Hall Institute of Medical Research, Australia

## Abstract

**Background:**

Characterizing infectious disease burden in Africa is important for prioritizing and targeting limited resources for curative and preventive services and monitoring the impact of interventions.

**Methods:**

From June 1, 2006 to May 31, 2008, we estimated rates of acute lower respiratory tract illness (ALRI), diarrhea and acute febrile illness (AFI) among >50,000 persons participating in population-based surveillance in impoverished, rural western Kenya (Asembo) and an informal settlement in Nairobi, Kenya (Kibera). Field workers visited households every two weeks, collecting recent illness information and performing limited exams. Participants could access free high-quality care in a designated referral clinic in each site. Incidence and longitudinal prevalence were calculated and compared using Poisson regression.

**Results:**

Incidence rates resulting in clinic visitation were the following: ALRI — 0.36 and 0.51 episodes per year for children <5 years and 0.067 and 0.026 for persons ≥5 years in Asembo and Kibera, respectively; diarrhea — 0.40 and 0.71 episodes per year for children <5 years and 0.09 and 0.062 for persons ≥5 years in Asembo and Kibera, respectively; AFI — 0.17 and 0.09 episodes per year for children <5 years and 0.03 and 0.015 for persons ≥5 years in Asembo and Kibera, respectively. Annually, based on household visits, children <5 years in Asembo and Kibera had 60 and 27 cough days, 10 and 8 diarrhea days, and 37 and 11 fever days, respectively. Household-based rates were higher than clinic rates for diarrhea and AFI, this difference being several-fold greater in the rural than urban site.

**Conclusions:**

Individuals in poor Kenyan communities still suffer from a high burden of infectious diseases, which likely hampers their development. Urban slum and rural disease incidence and clinic utilization are sufficiently disparate in Africa to warrant data from both settings for estimating burden and focusing interventions.

## Introduction

Most childhood deaths today occur in Sub-Saharan Africa, and over three-quarters of these deaths are due to infectious diseases [1]. Adults in Africa, who bear a disproportionate share of the world's HIV/AIDS epidemic, also have the highest mortality rates in the world [2]. Characterizing burden of infectious disease syndromes in Africa is important for targeting and prioritizing use of limited resources for optimal curative and preventive services and for research and development of novel strategies and interventions. Moreover, longitudinal surveillance of disease burden is important in monitoring impact of these public health expenditures. In Africa, the majority of cases of acute infectious illnesses do not present at health facilities [3], [4], and most deaths occur at home [5]. Community-based, household surveillance can supplement facility-based surveillance to define disease burden and measure impact of public health interventions. Nonetheless, few community-based studies of disease burden in Africa exist, and most that have been done took place before the widespread introduction of interventions that substantially reduced disease burden, such as insecticide-treated bednets, *Haemophilus influenzae* type b vaccine, improvements in access to safe drinking water, and wider use of cotrimoxazole and anti-retroviral medications in HIV-infected persons [6]–[9].

We describe comprehensive findings from two years of longitudinal, population-based surveillance at field clinics and specific households in geographically defined locations in rural western Kenya and an urban informal settlement in Nairobi for respiratory illness, diarrhea and acute febrile illness.

## Methods

### Ethical review

Written informed consent was obtained for data collection at the clinics and households. For minors, written informed consent was obtained from their parent or guardian. The protocol and consent forms were reviewed and approved by the Ethical Review Boards of the Kenya Medical Research Institute (KEMRI) (# 932) and the Institutional Review Board of the U.S. Centers for Disease Control and Prevention (CDC) (# 4566).

### Surveillance sites

The Kenyan International Emerging Infections Program of KEMRI/CDC has conducted population-based infectious disease surveillance (PBIDS) since late 2005 in two sites in Kenya: Asembo, a rural location in Nyanza Province in western Kenya along Lake Victoria and within the Kibera informal settlement in Nairobi (Table 1)[5], [10]–[13].

**Table 1:** Characteristics of study sites. Asembo, western Kenya and Kibera, Nairobi.

Characteristic	Asembo (years)	Kibera
Surveillance population, June 1, 2007, Total/<5 years	25,489/3,576	33,881/5,794
Area	100 km^2^	0.38 km^2^
Population density	325 persons per km^2^	77,000 persons per km^2^
Altitude in meters	1,100 meters	1,660 meters
Annual rainfall	1,358 mm*	558 mm[65]
Seasons	Long rains (March–May), short rains (Oct–Nov)	Long rains (March–May), short rains (Oct–Nov)
Average monthly temperature	24.5°C[65]	19.1°C average monthly temperature[65]
Breast-feeding	Supplemental feeding until 6 months, then wean	Supplemental feeding until 3–6 months, then wean
Malnutrition in children <5 years old	33% stunting, 7% wasting*	33% stunting, 8% wasting*
HIV prevalence adults/newborns	18.5%/3% *	15.0%/Not available*
Malaria endemicity	Holoendemic, year-round[5]	Not endemic, but imported cases common
Slept under insecticide-treated bednet last night (children <5)	76%* (2008)	N/A
Low birthweight births	15%[12]	N/A
Health facilities	3 first-level MOH clinics in surveillance site, and 3 more several km outside area	There are 5 registered non-MOH Health Centres within surveillance area and 8 other health facilities within 1 km to 2.5 km
Immunization coverage**	At 1 year, 79% DTP-HepB-Hib dose 3, 70% measles*(2007)	N/A
Occupation household head	Subsistence farming (65%), informal economy (13%), salaried (5%)	Salaried (53%), informal economy (43%)
Cooking fuel	Firewood (95%), charcoal (5%)*	Charcoal (76%), kerosene (22%), others (2%)*
Ethnic groups	Luo (>95%)[5]	Luo (>60%)*

*KEMRI/CDC unpublished data.

**Pneumococcal conjugate and rotavirus vaccines were not available in Kenya during this period. N/A – not available.

### Household surveillance

PBIDS household surveillance methods have been described previously [10]. Briefly, all households in both surveillance sites are offered enrollment. Eligible households are those located within 5 km and 1 km from the designated referral clinics for the project in Asembo and Kibera, respectively. Eligible persons must have resided permanently in these areas for 4 calendar months or be a child born to a woman enrolled in PBIDS. Enrollment was continuous in both sites since the project's beginning. Community interviewers visit enrolled households every two weeks (“fortnightly ” visits.) Participants are asked standardized questions, in local language, about recent illnesses. For certain key symptoms—cough, fever and diarrhea—the exact days of occurrence are recorded. For older children (approximately over 12 years old) and adults, interviews of that person are done. If not at home or unable to answer questions, a proxy who is knowledgeable about the participant's health is interviewed. For children unable to answer for themselves, the mother or other primary caretaker of the child is interviewed. Abbreviated physical exams are carried out on ill persons present during the visit, including axillary temperature, 1 minute respiratory rate, evaluation for lower chest wall indrawing and stridor in ill children, and observation for signs of dehydration. Community interviewers are secondary school graduates, who undergo extensive training on data collection and physical examination led by KEMRI/CDC clinicians, including WHO Integrated Management of Childhood Illness (IMCI) training videos [14].

### Clinic surveillance

Centrally located clinics were identified and enhanced in each surveillance area —in Asembo, St. Elizabeth Lwak Mission Hospital, operated by the Franciscan Sisters of St. Anna (henceforth referred to as “Lwak”), and in Kibera, Tabitha Clinic, operated by Carolina for Kibera (henceforth referred to as “Tabitha”)]. Enrolled participants receive free medical care at the study clinics for all acute, potentially infectious disease conditions, including syndromes not targeted for surveillance, by KEMRI/CDC-trained staff. Vital signs and symptoms are recorded by nurses or trained health facility recorders (secondary school graduates), and are verified by clinical staff. All temperatures are axillary and respiratory rates are measured for 1 minute using an audible countdown timer. Oxygen saturations are measured using fingertip pulse oximeters (NONIN Medical, Minnesota). Most examinations at both sites are carried out by clinical officers (similar to physician's assistants); two physicians at Tabitha Clinic also examined patients. Malaria blood smears are performed on all patients with history of fever or documented temperature ≥38.0°C. No x-rays were taken during the period described. Blood and stool for culture and combined nasopharyngeal and oropharyngeal swabs for PCR are collected from patients meeting clinical indications (data not presented). Lwak hospital has 40 inpatient beds and manages most hospitalizations, only referring complicated or surgical patients to the district hospital. Tabitha Clinic does not have inpatient capacity, and refers patients for hospitalization to a nearby district hospital.

Structured questionnaires detailing the current illness are completed for all sick visits at Lwak and Tabitha. At Lwak, scannable paper forms are used (TeleForm® software, Cardiff™, Vista, CA). Inconsistent or illogical data are returned to the clinic for correction. At Tabitha, data are entered directly into a custom-designed computerized system with internal quality control checks.

### Data analysis

Case definitions for infectious disease syndromes in the clinics are provided in Table 2. For clinic visits, incidence rates were calculated as the number of syndrome-specific visits per person-year of observation. Revisits for the same illness episode were not counted as separate episodes. For denominator calculations, permanent residence status on enrolled participants in the surveillance area was used to determine start and stop dates for person-time contribution. Adjusted rates of clinic visitation were calculated for each syndrome accounting for the percentage of all clinic visits made for each syndrome that went to the study clinics (i.e. Lwak, Tabitha) as opposed to other area clinics, as determined from the household visits. Calculating case-fatality proportions in the 30 days following a clinic visit was possible by longitudinal follow-up of all participants at the households.

**Table 2:** Case definitions for major infectious disease syndromes from household and clinic surveillance in Asembo, western Kenya (Lwak Hospital) and Kibera, Nairobi (Tabitha Clinic), June 1, 2006 – May 31, 2008.

Syndrome*	Clinic		Household	
	Children <5 years	Persons ≥5 years	Children <5 years	Persons ≥5 years
Acute respiratory infection (ARI)	(≥1 symptom): cough, difficulty breathing, chest pain, sore throat, sneezing, ear complaints or runny nose		cough or difficulty breathing	
Acute Lower Respiratory Infection (ALRI)	cough or difficulty breathing with one of the following: elevated respiratory rate for age** (non-severe pneumonia;) or IMCI danger sign***, lower chest wall indrawing, stridor, or oxygen saturation <90% (severe/very severe pneumonia) [14]	cough, difficulty breathing or chest pain and either documented axillary temperature ≥38.0°C or oxygen saturation <90% [40]	cough or difficulty breathing and rapid respiration for age** or chest indrawing noted on exam	cough or difficulty breathing or chest pain and documented temperature ≥38.0°C on exam
Diarrhea	≥3 looser than normal stools in a 24 hour period. Severe defined as IMCI danger sign or symptom/sign dehydration¶	≥3 looser than normal stools in a 24 hour period	≥3 looser than normal stools in a 24 hour period	
Acute Febrile Illness (AFI)	documented axillary temperature ≥38.0°C without an obvious cause, defined as cough, difficulty breathing, chest pain, signs of meningitis, or bloody diarrhea (positive malaria smear is not an exclusion)		report of fever, without evidence of another infection defined as cough or difficulty breathing or bloody diarrhea	

*Episodes of illness could meet more than one case definition (e.g. ARI and ALRI, ARI and diarrhea, ALRI and diarrhea.) AFI is mutually exclusive with ARI/ALRI and some types of diarrhea (i.e. bloody).

**Elevated respiratory rate for age based on WHO Integrated Management of Childhood Illness algorithm [14]; <2months, ≥60 breaths/minute; 2–11 months, ≥50 breaths/minute;12–59 months, ≥40 breaths/minute.

***IMCI danger signs are maternal report of convulsions, inability to drink or breastfeed, or vomiting everything, or on exam lethargy or unconsciousness. [14]

¶IMCI signs/symptoms of dehydration are the following: sunken eyes, slow skin pinch, restless/irritable behavior, drinking eagerly or not at all.[14]

For household visits, slightly modified case-definitions were used (Table 2). Because examination findings were part of the household definitions for ALRI, adjustments to the household ALRI rate were made for persons with cough or difficulty breathing who did not have an examination, including temperature, carried out at the household visit, assuming the same percentage would have ALRI as those who had cough or difficulty breathing and an exam done. Adjustments were made separately for each age group. Reported, rather than documented, fever was used to define AFI for household visits, as reported fever is commonly used as a diagnostic criterion for malaria in endemic areas and due to widespread use of antipyretics [14].

Although we asked about illness in the two weeks preceding home visit, we previously showed a significant decay of the recall of symptoms over that two week period, slightly impacted by age [10]. Therefore, to calculate the most accurate rates from the household visits avoiding decay of recall with time, we only used symptoms reported on the day of visit and the 3 previous days (days 0–3) for children <5 years and the day of visit and the 4 previous days (days 0–4) for persons ≥5 years, and adjusted the person-days calculations accordingly. Longitudinal prevalence rates were calculated as total number of days of a syndrome over total number of days of eligible observation, converted to person-years of observation. For calculation of longitudinal prevalence, the number of days of cough or difficulty breathing (ARI), diarrhea, and fever (AFI) in persons meeting the case definitions was used. Only days in which there was information as to whether the symptom was present or absent were included in denominators [10]. Prevalence rates for ALRI were not calculated because only cough, one component of the ALRI definition, was available for all days under surveillance. Incidence rates from household visits were calculated as the number of new episodes per person-year. We used a symptom-free interval for diarrhea of 3 days and for cough and fever of 7 days to define new episodes of the same syndrome [15], [16]. Denominators were calculated as for longitudinal prevalence, but, in addition, only days in which a new episode could be counted were included; therefore, subsequent days of an episode after the first day and the days during the symptom-free interval were excluded from the denominator [17].

For data collected from June 1, 2006 to May 31, 2008, we modeled incidence and longitudinal prevalence of illnesses at the household visits, and compared rural and urban rates, using Poisson regression (PROC GENMOD, SAS version 9.1, SAS Institute, Cary, North Carolina, USA). We controlled for clustering of symptoms at the household level using generalizing estimating equations (GEE). For the clinic visits, incidence rate ratios between rural and urban sites were calculated using Breslow's test for 95% confidence intervals (PEPI, Sagebrush publishers). For comparisons of adjusted rural and urban rates, the delta method was used [18].

We used incidence rates to project the number of cases of ALRI, diarrhea, and AFI in Nyanza Province and among the urban slum population in Nairobi, populations with similar epidemiology to our surveillance populations. We used 2007 population projections based on the 1999 national census, applying the same percentages of the national population that was residing in Nyanza and Nairobi Provinces, and assumed 60% of Nairobi residents were residents of informal settlements [19]–[21]. Because our surveillance sites were likely not representative of other provinces of Kenya in terms of certain risk factors (e.g., HIV and malaria prevalence), we did not make national projections during this analysis.

## Results

### General morbidity

There were 20,830 visits to the referral clinic in Asembo and 38,857 visits to Kibera during the study period, yielding overall rates of clinic visitation of 0.43 per person-year and 0.69 visits per person-year, respectively [Rate Ratio (RR) - 0.63, 95% CI 0.62–0.64]. Clinic visit rates were highest in children <5 years old (0.77 visits/person-year in Asembo, 2.4 visits/person-year in Kibera.) Clinic visit rates were higher for all age groups <35 years in Kibera than Asembo, similar in persons 35–49 years and higher in Asembo for persons ≥50 years.

During the study period, 1,181,788 interviews of household members (or proxies) were completed in Asembo and 710,997 in Kibera. For children <5 years, illness episodes were reported during the last two weeks in 40.3% of household visits in Asembo compared with 13.7% in Kibera (RR = 2.9, 95% CI 2.9–3.0). For persons ≥5 years, illness episodes were reported during the last two weeks in 28% of household visits in Asembo compared with 5.1% in Kibera (RR = 5.4, 95% CI 5.4–5.5).

### Acute Respiratory Infections

ARI was the most common syndrome in both sites (Table 3). ARI rates were highest in young children, with cough or difficulty breathing being reported for almost two full months per year in Asembo and one month per year in Kibera for children <5 years old. ARI incidence was 5.6 times higher at the household visit than at the clinic for children in Asembo and 5.1 times higher for adults. In contrast, in Kibera the incidence detected for ARI in the household was 0.89 times and 0.71 times the incidence in the clinic for children and adults, respectively.

**Table 3:** Rates of Acute Respiratory Illness by age group from household and clinic surveillance in Asembo, western Kenya (Lwak Hospital) and Kibera, Nairobi (Tabitha Clinic), June 1, 2006 – May 31, 2008.

	Household visits						Clinic visits					
	Longitudinal Prevalence			Incidence			Crude Incidence			Adjusted Incidence*		
Age	Asembo	Kibera	RR (95% CI)	Asembo	Kibera	RR (95% CI)	Asembo	Kibera	RR (95% CI)	Asembo	Kibera	RR (95% CI)
<1 year	69.0	39.4	1.8 (1.7–1.9)	9.53	4.90	2.0 (1.8–2.1)	0.80	2.47	0.32 (0.30–0.34)	1.83	5.89	0.31 (0.29–0.33)
12–23 mo.	63.6	29.5	2.2 (2.0–2.3)	8.60	3.61	2.4 (2.2–2.6)	0.65	1.78	0.38 (0.35–0.40)	1.39	3.81	0.37 (0.34–0.39)
24–59 mo.	55.9	21.7	2.6 (2.4–2.7)	7.21	2.76	2.6 (2.5–2.8)	0.62	1.24	0.50 (0.48–0.52)	1.27	3.24	0.39 (0.38–0.41)
<5 years	59.9	26.8	2.2 (2.2–2.3)	7.92	3.33	2.4 (2.3–2.5)	0.67	1.55	0.43 (0.42–0.45)	1.42	3.76	0.38 (0.37–0.39)
5–17 years	31.2	7.70	4.1 (3.8–4.3)	3.58	0.90	4.0 (3.7–4.2)	0.31	0.37	0.83 (0.80–0.86)	0.61	1.25	0.49 (0.47–0.51)
18–34 years	25.0	6.59	3.8 (3.6–4.0)	2.49	0.66	3.8 (3.5–4.0)	0.26	0.28	0.92 (0.88–0.96)	0.56	0.98	0.57 (0.55–0.60)
35–49 years	35.2	8.04	4.4 (4.0–4.8)	3.10	0.74	4.2 (3.8–4.6)	0.40	0.32	1.3 (1.2–1.3)	0.88	1.11	0.79 (0.74 –0.84)
≥50 years	61.9	7.66	8.1 (7.0–9.3)	4.36	0.70	6.3 (5.3–7.4)	0.33	0.20	1.7 (1.5–1.9)	0.77	0.74	1.0 (0.94–1.1)
Total ≥5 years	35.8	7.26	4.9 (4.7–5.1)	3.38	0.77	4.4 (4.2–4.6)	0.31	0.31	0.99 (097–1.0)	0.66	1.08	0.61 (0.60–0.63)

*Adjusted clinic rates were calculated by extrapolating from those with same syndrome defined at household visit who sought care at a clinic besides the designated referral clinics, Lwak and Tabitha (see methods). In Asembo, in the age categories <1 year, 12–23 months, 24–59 months, <5 years, 5–17 years, 18–34 years, 35–49 years, ≥50 years and ≥5 years, the percentage of all clinic visits for ARI to Lwak were 44%, 47%, 49%, 47%, 50%, 46%, 45%, 43%, and 47%, respectively. For Kibera, for the same age groups, the percentages of all clinic visits for ARI to Tabitha were 42%, 46%, 38%, 41%, 30%, 29%, 29%, 27% and 29%, respectively.

Longitudinal prevalence is days of cough or difficulty breathing as part of an ARI episode per person-year. Incidence is number of episodes of ARI per person-year.

#### Acute lower respiratory tract infections

The rate of ALRI resulting in clinic visitation was lower in Asembo than Kibera among children <5 years old (Table 4). Among children <5 years old, 0.41% and 0.89% of ALRI episodes in the clinic occurred in neonates in Asembo and Kibera, respectively. Persons ≥5 years had much lower rates of ALRI than children in both Asembo and Kibera. In contrast to all other syndromes, ALRI rates were higher in the clinic than in the household. Among children with ALRI seen in the clinic, 8.5% in Asembo and 2.0% in Kibera had auscultatory wheezing reported on exam at the clinic. Among children with ALRI in the clinic, 56% in Asembo and 42% in Kibera met IMCI criteria for severe or very severe pneumonia (Table 4). In Asembo, 3% of children with ALRI had oxygen saturation <90% and only 1% had oxygen saturation <90% without other criteria for IMCI pneumonia of any severity; 7.5% of adults with ALRI had oxygen saturation <90%. In Kibera, 11% of children with ALRI had oxygen saturation <90% but none had oxygen saturation <90% without other criteria for IMCI pneumonia of any severity; 23% of adults with ALRI had oxygen saturation <90%. At Lwak Hospital, 64% of children and 34% of persons ≥5 years with ALRI were hospitalized.

**Table 4:** Rates of Acute Lower Respiratory Illness by age group from household and clinic surveillance in Asembo, western Kenya (Lwak Hospital) and Kibera, Nairobi (Tabitha Clinic), June 1, 2006 – May 31, 2008.

	Household visits			Clinic visits					
	Incidence*			Crude Incidence			Adjusted Incidence**		
Age	Asembo	Kibera	RR (95% CI)	Asembo	Kibera	RR (95% CI)	Asembo	Kibera	RR (95% CI)
<1 year	0.65	0.34	1.9 (1.5–2.5)	0.20	0.67	0.29 (0.26–0.33)	0.44	0.87	0.51 (0.45–0.57)
12–23 mo.	0.51	0.34	1.5 (1.2–2.0)	0.21	0.62	0.34 (0.31–0.39)	0.47	0.74	0.63 (0.56–0.71)
24–59 mo.	0.11	0.093	1.2 (0.90–1.7)	0.13	0.26	0.50 (0.46–0.55)	0.26	0.31	0.83 (0.76–0.92)
<5 years	0.29	0.19	1.5 (1.3–1.8)	0.16	0.41	0.40 (0.37–0.42)	0.36	0.51	0.70 (0.66–0.75)
5–17 years	0.03	0.009	3.6 (2.4–5.4)	0.043	0.036	1.2 (1.1–1.3)	0.083	0.045	1.9 (1.7–2.1)
18–34 years	0.01	0.005	2.5 (1.4–4.5)	0.025	0.012	2.1 (1.8–2.5)	0.051	0.016	3.2 (2.7–3.8)
35–49 years	0.02	0.008	2.7 (1.3–5.7)	0.039	0.014	3.0 (2.3–3.9)	0.082	0.019	4.3 (3.4–5.6)
≥50 years	0.014	0	NA	0.029	0.010	2.92 (1.9–4.4)	0.068	0.015	4.6 (3.1–7.0)
Total ≥5 years	0.02	0.007	3.3 (2.5–4.5)	0.033	0.020	1.7 (1.5–1.8)	0.067	0.026	2.6 (2.4–2.8)

*Rates from household visits for ALRI are adjusted by the percentage of persons with reported cough or difficulty breathing who did not have an exam done at home (see methods). In Asembo, in the age categories <1 year, 12–23 months, 24–59 months, <5 years, 5–17 years, 18–34 years, 35–49 years, ≥50 years and ≥5 years, these percentages were 29%, 33%, 46%, 39%, 72%, 41%, 33%, 24%, and 49%, respectively. For Kibera, for the same age groups, the percentage without an exam were 36%, 33%, 43%, 38%, 60%, 21%, 30%, 37%, and 40%, respectively.

**Adjusted clinic rates were calculated by extrapolating from those with same syndrome defined at household visit who sought care at a clinic besides the designated referral clinics, Lwak and Tabitha (see methods). In Asembo, in the age categories <1 year, 12–23 months, 24–59 months, <5 years, 5–17 years, 18–34 years, 35–49 years, ≥50 years and ≥5 years, the percentage of all clinic visits for ALRI to Lwak were 45%, 45%, 51%, 46%, 51%, 49%, 48%, 43%, and 49%, respectively. For Kibera, the percentage of all clinic visits for ALRI to Tabitha 77%, 84%, 84%, 82%, 81%, 76%, 69%, 68%, and 77%, respectively.

Incidence is number of episodes of ALRI per person-year.

#### Diarrhea

Rates for diarrhea resulting in clinic visit were lower in Asembo than Kibera among children <5 years (Table 5). Among children <5 years old, 0.42% and 0.37% of diarrhea episodes in the clinic occurred in neonates in Asembo and Kibera, respectively. On average, children <5 years had 9.5 and 8.1 diarrhea days per year in Asembo and Kibera, respectively. Persons ≥5 years had much lower rates of diarrhea than children in both Asembo and Kibera. Numbers of diarrhea episodes as reported in the household were 6-fold and 2-fold higher than those seen in the referral clinics in Asembo and Kibera, respectively. Among children with diarrhea visiting the referral clinics, 40% in Asembo and 11% in Kibera had a sign of dehydration or an IMCI danger sign. At Lwak, 26% of children and 20% of persons ≥5 years with diarrhea were hospitalized.

**Table 5 pone-0016085-t005:** Rates of diarrhea by age group from household and clinic surveillance in Asembo, western Kenya (Lwak Hospital) and Kibera, Nairobi (Tabitha Clinic), June 1, 2006 – May 31, 2008.

	Household visits						Clinic visits					
	Longitudinal Prevalence			Incidence			Crude Incidence			Adjusted Incidence*		
Age	Asembo	Kibera	RR (95% CI)	Asembo	Kibera	RR (95% CI)	Asembo	Kibera	RR (95% CI)	Asembo	Kibera	RR (95% CI)
<1 year	16.1	11.8	1.4 (1.2–1.5)	3.25	1.88	1.7 (1.5–2.0)	0.35	0.96	0.36 (0.33–0.40)	0.73	1.28	0.57 (0.52–0.63)
12–23 mo.	17.3	12.9	1.3 (1.2–1.5)	4.04	2.32	1.7 (1.6–2.0)	0.24	0.79	0.31 (0.28–0.34)	0.53	0.93	0.57 (0.52–0.64)
24–59 mo.	4.9	5.1	0.96 (0.86–1.1)	1.29	0.98	1.3 (1.2–1.5)	0.10	0.39	0.26 (0.23–0.29)	0.21	0.47	0.45 (0.40–0.49)
<5 years	9.5	8.1	1.2 (1.2–1.3)	2.19	1.43	1.5 (1.4–1.7)	0.19	0.57	0.33 (0.31–0.34)	0.40	0.71	0.56 (0.53–0.60)
5–17 years	1.2	0.66	1.7 (1.5–1.9)	0.35	0.15	2.4 (2.3–2.7)	0.031	0.059	0.53 (0.47–0.58)	0.066	0.071	0.93 (0.84–1.03)
18–34 years	1.8	0.52	3.5 (3.1–4.0)	0.44	0.11	4.0 (3.5–4.6)	0.047	0.039	1.2 (1.1–1.4)	0.095	0.054	1.8 (1.6–2.0)
35–49 years	2.8	0.73	3.8 (3.2–4.6)	0.70	0.15	4.6 (3.9–5.5)	0.071	0.051	1.4 (1.2–1.6)	0.14	0.079	1.8 (1.5–2.1)
≥50 years	3.8	0.73	5.3 (4.0–6.9)	1.07	0.17	6.2 (4.7–8.2)	0.051	0.040	1.3 (1.0–1.6)	0.11	0.053	2.1 (1.7–2.6)
Total ≥5 years	2.0	0.61	3.3 (3.0–3.5)	0.55	0.13	4.1 (3.8–4.5)	0.043	0.047	0.92 (0.87–0.98)	0.090	0.062	1.5 (1.4–1.6)

*Adjusted clinic rates were calculated by extrapolating from those with same syndrome defined at household visit who sought care at a clinic besides the designated referral clinics, Lwak and Tabitha (see methods). In Asembo, in the age categories <1 year, 12–23 months, 24–59 months, <5 years, 5–17 years, 18–34 years, 35–49 years, ≥50 years and ≥5 years, the percentage of all clinic visits for diarrhea to Lwak were 47%, 45%, 48%, 47%, 47%, 49%, 50%, 47%, and 48%, respectively. For Kibera, for the same age groups, the percentage of all clinic visits for diarrhea to Tabitha were 75%, 85%, 82%, 81%, 84%, 73%, 65%, 76%, and 76%, respectively.

Longitudinal prevalence is days of diarrhea per person-year. Incidence is number of episodes of diarrhea per person-year.

#### Acute Febrile Illness

In contrast to the other 3 syndromes, clinic visitation rates for AFI were higher in Asembo than Kibera among children <5 years, likely due to greater malaria prevalence in rural western Kenya (Table 6). Among children <5 years old, 0.34% and 0.42% of AFI episodes in the clinic occurred in neonates in Asembo and Kibera, respectively. On average, children <5 years had 37 and 11 fever days per year in Asembo and Kibera, respectively. Persons ≥5 years had much lower rates of AFI than children in both Asembo and Kibera. Among children with AFI in the clinic who had a blood smear done, 68% in Asembo and 23% in Kibera had malaria parasites seen. At Lwak Hospital, 40% of children and 28% of persons ≥5 years with AFI were hospitalized.

**Table 6:** Rates of acute febrile illness (AFI) by age group from household and clinic surveillance in Asembo, western Kenya (Lwak Hospital) and Kibera, Nairobi (Tabitha Clinic), June 1, 2006 – May 31, 2008.

	Household visits						Clinic visits					
	Longitudinal Prevalence			Incidence			Crude Incidence			Adjusted Incidence*		
Age	Asembo	Kibera	RR (95% CI)	Asembo	Kibera	RR (95% CI)	Asembo	Kibera	RR (95% CI)	Asembo	Kibera	RR (95% CI)
<1 year	45.2	17.9	2.5 (2.4–2.7)	12.0	4.2	2.9 (2.6–3.1)	0.046	0.092	0.50 (0.38–0.66)	0.11	0.12	0.92 (0.70–1.21)
12–23 mo.	42.1	14.1	3.0 (2.8–3.2)	11.4	3.4	3.4 (3.1–3.7)	0.080	0.087	0.92 (0.74–1.2)	0.17	0.11	1.6 (1.3–2.0)
24–59 mo.	32.8	8.1	4.0 (3.8–4.3)	8.9	1.9	4.6 (4.3–4.9)	0.088	0.062	1.4 (1.2–1.7)	0.18	0.076	2.4 (2.1–2.8)
<5 years	37.0	11.3	3.3 (3.1–3.4)	9.9	2.7	3.8 (3.6–3.9)	0.077	0.073	1.1 (0.94–1.2)	0.17	0.090	1.9 (1.7–2.1)
5–17 years	15.1	2.5	6.1 (5.7–6.4)	3.9	0.57	6.7 (6.3–7.2)	0.022	0.020	1.1 (0.94–1.3)	0.047	0.026	1.9 (1.6–2.1)
18–34 years	12.6	2.8	4.6 (4.3–4.9)	2.7	0.54	4.9 (4.6–5.2)	0.0085	0.007	1.2 (0.94–1.6)	0.019	0.010	1.9 (1.5–2.5)
35–49 years	20.5	3.8	5.4 (5.0–5.9)	3.7	0.72	5.2 (4.7–5.7)	0.007	0.007	1.0 (0.65–1.6)	0.016	0.011	1.5 (0.98–2.4)
≥50 years	31.1	3.4	9.2 (7.8–10.9)	4.3	0.58	7.3 (6.2–8.7)	0.0031	0.002	1.6 (0.61–4.0)	0.008	0.0029	2.7 (1.01–7.1)
Total ≥5 years	18.1	2.8	6.4 (6.1–6.7)	3.6	0.58	6.2 (6.0–6.5)	0.013	0.011	1.1 (0.96–1.2)	0.030	0.015	1.8 (1.6–2.0)

*Adjusted clinic rates were calculated by extrapolating from those with same syndrome defined at household visit who visited a clinic besides the designated referral clinics, Lwak and Tabitha (see methods). In Asembo, in the age categories <1 year, 12–23 months, 24–59 months, <5 years, 5–17 years, 18–34 years, 35–49 years, ≥50 years and ≥5 years, the percentage of all clinic visits for AFI to Lwak were 42%, 47%, 48%, 46%, 46%, 44%, 44%, 40%, and 44%, respectively. For Kibera, for the same age groups, the percentage of all clinic visits for AFI to Tabitha were 78%, 83%, 82%, 81%, 78%, 70%, 67%, 69%, and 73%, respectively.

Longitudinal prevalence is days of fever as part of an AFI per person-year. Incidence is number of episodes of AFI per person-year.

#### Mortality

**Figure 1:**
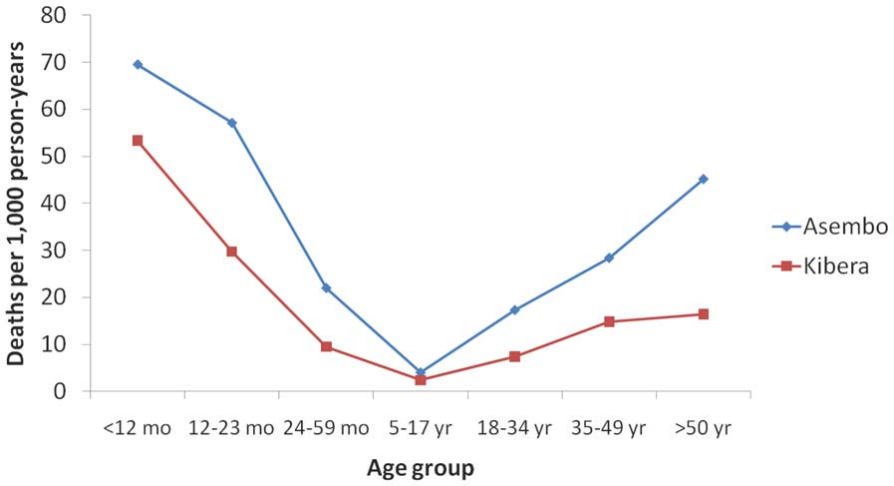
Overall mortality rates by age for Asembo and Kibera in areas of population-based surveillance.

For illness episodes presenting at the clinic, the case-fatality ratio was higher for all illnesses for children and adults in Asembo than Kibera (Table 7). Overall, mortality rates were also higher for all groups in Asembo than Kibera, particularly at the extremes of age (Figure 1)

**Table 7:** Case-fatality ratio (# deaths/# cases) by syndrome, within 30 days of clinic visit date.

	ALRI	Diarrhea	AFI
	Asembo (for all patients/for admitted patients)		
<5 years	1.7%/2.6%	1.5%/4.6%	0.51%/1.3%
≥5 years	2.1%/5.1%	2.8%/9.9%	0.55%/2.0%
	Kibera (for all patients)		
<5 years	0.23%	0.19%	0.13%
≥5 years	0.31%	0.22%	0.37%

#### Burden projections

The projected number of episodes of illness in 2007 was substantial in both Nyanza province (population of 4.3 million in 1999) and the informal settlements of Nairobi, ranging up to almost 27 million episodes of AFI in Nyanza Province (Table 8).

**Table 8:** Number of episodes of ALRI, diarrhea and AFI estimated to have occurred in Nyanza Province and informal Nairobi settlements in 2007 based on household (HH)- and clinic-based surveillance, Kenya.

	Nyanza Province		Nairobi informal settlements	
	<5 years	≥5 years	<5 years	≥5 years
2007 Projected population	980,089	4,679,199	336,463	1,606,363
ALRI: Incidence* (HH/Clinic)Episodes (HH/Clinic)	0.29/0.36284,226/352,832	0.02/0.06793,584/313,506	0.19/0.5163,928/145,011	0.007/0.02611,245/35,294
Diarrhea: Incidence* (HH/Clinic)Episodes (HH/Clinic)	2.19/0.402,146,396/392,035	0.55/0.092,573,560/421,128	1.43/0.71481,143/201,878	0.13/0.062208,827/84,164
AFI: Incidence* (HH/Clinic)Episodes (HH/Clinic)	9.9/0.179,702,886/166,615	3.6/0.0316,845,117/140,376	2.7/0.09908,451/25,590	0.58/0.015931,691/20,362

*Incidence is number of episodes per person-year from tables 4–6.

## Discussion

Our longitudinal, population-based surveillance provides one of the few recent, comprehensive estimates of infectious disease burden in Africa, being unique in measuring burden for multiple infectious disease syndromes using common methodology in both rural and urban settings among children and adults, and in surveying at both clinics and in the household. Household visits served two purposes. First, they were used to encourage ill participants to access the referral clinic. Second, household visits were useful in interpreting and adjusting incidence rates. Shops and drug-sellers are more common sources of healthcare than licensed clinics in both areas [22], [23]. Multiple factors influence healthcare utilization at clinics, including cost, distance, quality of care, and severity of syndrome [24]–[27]. Even when offering free high-quality care as part of surveillance, many ill persons did not access our designated referral clinics, particularly in the rural area, where only 30–40% of ill participants sought care at any clinic and only a half of these at the project's designated referral clinic, percentages that did not vary substantially during the surveillance period [28]. Rates of ARI, diarrhea and AFI, but not ALRI, were higher when calculated from household visits than from clinic data. The higher rates of ALRI in the clinic than the household is likely due to differences in the case definitions used in the clinic and household, as well as a higher percentage of persons with ALRI visiting clinics due to the greater severity of illness than the other syndromes. The smaller difference between clinic and household rates of disease in Kibera than Asembo is because healthcare utilization overall, and particularly to the designated referral clinic, was higher in the urban site [22], [23], [28]. Distance of residence to clinic clearly influences health utilization [26]; those distances are much less in Kibera. In addition it is possible that people living in Kibera are more educated and comfortable with use of licensed clinic facilities than residents in Asembo, a remote rural area. Nonetheless, our findings suggest that a substantial burden of infectious disease illness does not present to the clinic, especially in rural Africa, highlighting the need for preventive measures (e.g. vaccines, insecticide-treated bednets) and non-clinic-based case management strategies in such settings [29]–[34].

Community-based studies of clinically-defined ALRI in African children have yielded varied incidences, with our estimates (0.29 per person-year rural, 0.19 per person-year urban) falling in the middle of the range when compared with peri-urban Nigeria (1.3 episodes/person-year), rural Gambia (0.45 episodes/person-year), urban Nigeria (0.08–0.22 episodes/person-year), rural central Kenya (0.21 episodes/person-year), and rural Ghana (0.06 episodes/person-year) [8], [9], [35]–[38]. The reason for higher rates of wheezing in children in the rural area when compared with urban in our study might be related to greater exposure to indoor air pollution from cooking with firewood in the rural area (Table 1), but could also be related to the frequency of certain viral infections (e.g. RSV) [39]. For ARI in children, the incidence we found (7.9 episodes/person-year rural, 3.5 episodes/person-year urban) is similar to rates from other community-based African studies – rural Kenya, 6.6 episodes/person-year, urban Nigeria 8.1 episodes/person-year [9], [37], [38].

While facility-based data on respiratory illness in adults exist, there have been no community-based data [40]–[45]. The facility-based rates of ALRI we estimate of 67 and 26 per 1,000 person-years in rural and urban Kenya, respectively, are on the higher end of estimates found in other studies in Africa, being more comparable with rates in HIV-infected populations (37–69 per 1000) [40], [43], [45]. Among HIV-negative or HIV-status unknown adult populations in Africa, ALRI rates have ranged from 2–10 per 1,000 [40], [41], [44], [46], [47]. There are several reasons likely responsible for the somewhat higher rate of adult ALRI we found. First, both our populations have high rates of adult HIV infection (15–18%, KEMRI/CDC data.) Second, we used a clinical ALRI definition that might have captured upper respiratory and influenza-like illness, in addition to ALRI [48]. Third, we collected longitudinal data in relatively small populations, and provided free, high-quality healthcare, which could have led to higher levels of health facility utilization than in other African studies.

The incidence rate of diarrhea we found in children of 1.9–2.2 episodes per child-year is lower than other estimates in Africa from the 1980s [7]. In a study from urban Nairobi, among children <3 years, 3.5 diarrheal episodes per year were recorded, whereas in Guinea-Bissau and Zaire 10.4 and 6.3 episodes per year, respectively, were recorded in children <5 years [7], [49]–[51]. It is possible that in the two decades since most earlier studies were undertaken that diarrhea disease rates have decreased in Africa due to interventions such as safe-water promotion, better hygiene, increased measles vaccination coverage, and reduced malnutrition [6]. Alternatively, differences in methodology, such as frequency of visits, or cultural concepts about what constitutes “diarrhea” could have contributed. Few community-based studies estimate diarrhea rates in African adults. In rural Uganda during weekly household visits, HIV-positive persons ≥5 years not on cotrimoxazole prophylaxis had 194 diarrheal episodes per 100 person-years, while HIV-negative persons ≥10 years living in the same households experienced 15.5 episodes per 100 person-years [52], [53]. We found diarrhea rates in adults (rural, 55 per 100 person-years; urban, 13 per 100 person-years) closer to the HIV-negative Ugandan population.

Rates of AFI are not usually calculated in African settings, despite fever usually being the most common presenting symptom [54]–[57]. More often, rates of specific etiologies of febrile illness are reported. Invasive bacterial disease rates among children range from 505 to 1,100 episodes per 100,000 person-years in various African settings [35], [58], [59]. These were estimates from hospital-based studies, and likely underestimate the true rate. A majority (up to 80%) of malaria illness never gets diagnosed in the community because of low levels of healthcare seeking and lack of laboratory testing [60]. Estimates of malaria rates in children are obtained from control groups during recent studies of candidate malaria vaccines, where symptomatic malaria incidence ranged from 17–26 per 100 person-years [29], [33]. We found clinic rates of AFI among children that were lower than the symptomatic malaria incidence rates from these trials, but higher than the invasive bacterial disease rates. At the household visits, we found AFI incidence was several-fold higher, with children experiencing 10 febrile episodes a year in the rural site and 3 in the urban site.

Among adult African populations, even less incidence data on AFI are available. From control arms of clinical trials in malaria-endemic areas of western Kenya, incidence rates of symptomatic and asymptomatic malaria parasitemia were 1–2 episodes per person-year [61], [62]. One review estimated the typhoid rate to be 39/100,000 in East Africa [54]. The incidence of AFI defined in the household for persons ≥5 years in our surveillance was several-fold higher than either these malaria or typhoid rates, suggesting that these diseases, particularly malaria, may be under-diagnosed and, perhaps more importantly, broader diagnostic testing is needed to elucidate the multiple causes of fever in older children and adults in Africa.

The mortality pattern in our surveillance areas, with highest rates among young children then rising again in young adults, is typical of parts of Africa with high levels of HIV [1], [2]. Mortality and case-fatality ratios following clinic visits were higher in Asembo than Kibera. There are several potential reasons for this difference. First, Tabitha clinic sees more patients than Lwak Hospital, likely reflecting greater healthcare use for milder illness [26]. Because of this, illness episodes might be treated at an earlier stage preventing progression to severe disease, which in turn would result in lower overall mortality. Second, more challenging access to quality health care (because of distance and poor roads, especially during rainy seasons) might have resulted in later presentation of illness in the rural setting. Third, some diseases, particularly malaria, are more prevalent in the rural area. Lastly, there might have been surveillance bias. Many residents of Kibera consider their ancestral home to be elsewhere in Kenya, and it is culturally important to be home for death and burial; thus, it is possible that pre-terminal patients would go “up-country” to die, and not be reported or counted as deaths in the Kibera surveillance system.

Besides incidence, we also report on longitudinal prevalence from the household visits, which is the cumulative number of days over a given time period in which a person is ill [63], [64]. Longitudinal prevalence might be a better measure of illness burden, because unlike incidence, it accounts for both the number and duration of illness episodes. Longitudinal prevalence has been more closely correlated with growth faltering and mortality in children with diarrhea than has incidence [63], [64]. Longitudinal prevalence of fever and lower respiratory tract infections in children has also been associated with growth faltering, but has not been evaluated as a disease burden measure in adults [65].

We encountered several challenges when comparing rates of disease in the clinic and the community. The specificity of the case definitions for the same syndrome likely differed for household- and clinic-based surveillance. While trained field workers evaluated participants in the household visits, clinicians did so in the clinics, where they could verify reported symptoms based on their clinical assessment of the patient. Also, patients were likely to be sicker when seen in the clinic than at the household visit and so had a greater prior probability of having the syndromes under surveillance. Our decisions whether to use reported versus documented findings was based on previously used diagnostic criteria [8], [14]. While reported symptoms in the household might have had reduced specificity (e.g. AFI), using an exam-based case definition in the household introduced other complications because a person must be at home and acutely ill at the time of the biweekly household visit to capture abnormal signs. In using an exam-based definition at the household for ALRI, we adjusted rates to compensate for individuals who might have had ALRI who did not have an exam done, an adjustment that could have been prone to bias if those without exams were more or less likely to have ALRI than those with exam.

We adjusted clinic rates to avoid underestimation of the true burden that would result from basing calculations on only one clinic in sites where multiple clinics were available to the population. We assumed that those illnesses episodes in which care was sought at another local clinic were likely to be of equivalent severity. Similar adjustments to incidence rates based on persons not sampled have been made before for estimates of acute febrile illness in Egypt [55]. It is possible though that those illness episodes that resulted in a visit to the referral clinic were not the same as those that went to other clinics. In Asembo, the bias might have been for more severe illnesses to visit Lwak, where people knew admission would be free. Whereas in Kibera, the opposite might have been the case, whereby ill persons who felt they would need hospitalization would bypass Tabitha and go directly to an inpatient hospital.

In conclusion, disease burden estimates such as this derived from local surveillance data can provide credible data to decision makers about the need for targeted interventions and what health and economic impact such interventions might yield. We demonstrated that disease rates in the community are higher in rural than urban Kenya, although clinic-based rates tend to be higher in the urban setting, where health utilization is better. We also showed that in rural Kenya many illness episodes are missed at the clinic and the burden of infectious diseases is likely several-fold higher than can be measured at the clinic. Such data suggest interventions to improve health care access or to implement non-facility based treatment strategies, such as effective home-management of malaria and diarrhea, might have a large public health impact in rural Africa [30]. Both communities had an intolerable burden of illness, with children experiencing illness in up to one-quarter of the year. Proven interventions, such as insecticide-treated bednets and the pneumococcal conjugate and rotavirus vaccines, are urgently needed to alleviate this burden [31], [32], [34]. Without more effective interventions to control these common infectious disease syndromes, continued high morbidity and mortality in poor African settings will continue to hamper development of these communities.
